# MicroRNA-205 acts as a tumor suppressor in osteosarcoma via targeting RUNX2

**DOI:** 10.3892/or.2021.8106

**Published:** 2021-06-07

**Authors:** Can Zhang, Feng Long, Jun Wan, Yihe Hu, Hongbo He

Oncol Rep 35: 3275-3284, 2016; DOI: 10.3892/or.2016.4700

Following the publication of this paper, it was drawn to the Editors attention by a concerned reader that certain of the cell Transwell assay data in the article (featured in Fig. 3C and D) were strikingly similar to data that appearing in different form in other articles by different authors at different research institutions, which were already under consideration for publication or had already been published elsewhere at the time of the present articles submission.

Owing to the fact that the contentious data in the above article had already appeared in different form in other articles prior to its submission to *Oncology Reports*, the Editor has decided that this paper should be retracted from the Journal. After having been in contact with the authors, they agreed with the decision to retract the paper. The Editor apologizes to the readership for any inconvenience caused.

